# Cell Wall-Formed Dietary Fiber in Tartary Buckwheat Governs Selective Enrichment of Phytochemicals to Enhanced Postprandial GLP-1 Response

**DOI:** 10.3390/ijms27219470

**Published:** 2026-10-23

**Authors:** Menglin Jiang, Yao Chen, Genyi Zhang

**Affiliations:** State Key Laboratory of Food Science and Resources, School of Food Science and Technology, Jiangnan University, Wuxi 214122, China

**Keywords:** whole grain foods, cell wall-formed dietary fiber (CW-DF), phytochemical, selective enrichment, glucagon-like peptide 1 (GLP-1)

## Abstract

Glucagon-like peptide-1 (GLP-1) is well-known for its biological functions in glucose homeostasis which aligns closely with the antidiabetic function of whole grain foods (WGFs). Although postprandial GLP-1 responses to WGF have been extensively studied, the influence of cell wall-formed dietary fiber (CW-DF), as the main functional component of WGFs, on the GLP-1 response remains poorly understood. Here, Tartary buckwheat flour fractions with different particle sizes (small particle size (SF: 0.2 mm), middle particle size (MF: 0.6 mm), and large particle size (BF: 0.9 mm)), reflecting different degrees of cell wall integrity, were used to investigate how particle size modulates GLP-1 secretion. The in vivo mouse study showed that intact endosperm cells from Tartary buckwheat grains significantly increased the postprandial GLP-1 response. Further study demonstrated that only the hydrolysate from BF augmented GLP-1 secretion from enteroendocrine STC-1 L-cells, which is all positively correlated with the selectively enriched phytochemicals in BF particles (ononin, ginkgolide C, pristimerin, and pentadecanoic acid). Significant correlations between particle size, phytochemical profiles and GLP-1 release demonstrated that the integrity of CW-DF is essential to the nutritional properties of WGFs. Molecular docking simulations suggested putative interactions between phytochemicals and bitter taste receptors in GLP-1 secretion. Thus, cell wall integrity is a pivotal regulator of the health benefits of WGFs, offering a mechanistic rationale for minimal processing to harness the health benefits of WGFs through targeted endogenous GLP-1 production.

## 1. Introduction

Glucagon-like peptide 1 (GLP-1), an intestinal hormone secreted by enteroendocrine L-cells upon stimulation by nutrients within the intestinal lumen, is widely acknowledged for its antidiabetic and anti-obesity bioactivities. Apart from these metabolic functions, GLP-1 additionally mediates neuroprotective, antibacterial and anti-inflammatory effects while supporting cardiovascular wellness. Multiple studies have demonstrated a positive correlation between whole-grain consumption and elevated GLP-1 concentrations. To illustrate, biscuits made from flaked sorghum raise postprandial GLP-1 concentrations and extend satiety duration [1]. Polyphenols from whole millet grains also facilitate endogenous GLP-1 production [2]. Intake of rye bread likewise augments postprandial GLP-1 responses [3]. Substituting 85% of wheat flour with broken grain kernels in bread formulations distinctly amplified GLP-1 secretion [4]. Considering the shared physiological advantages of whole grain foods and GLP-1, the induction of GLP-1 secretion is likely a pivotal mechanism through which whole grains exert their health-improving properties.

The interactions between dietary fibers and phenolic compounds—key bioactive components in whole grain foods (WGFs)—significantly contribute to the health benefits of WGFs. Studies have demonstrated that phenolic compounds can selectively bind to cell-wall polysaccharides [5] whereas polysaccharide morphology (surface properties and porosity), their chemical composition, and molecular structure all influence their selective binding [6]. Regarding GLP-1 secretion mechanisms, both metabolites from fiber fermentation, such as short-chain fatty acids (SCFAs) [7], and phenolic compounds [8] have been known to be potent stimulants of GLP-1 secretion. In WGFs, while the compositional complexity of whole grains is well-documented, the relationship between the physical structure (particularly cell wall-sourced fibers) of WGFs and GLP-1 secretion remains poorly understood [9]. This knowledge gap underscores the need for a holistic approach to investigate the relationship between WGFs’ physical properties and GLP-1 secretion profiles. Milled whole grain flour particles represent an ideal holistic model system for such investigations, as they not only preserve the native interactions between components but also have varying physical attributes (morphology, porosity, and size) that are intimately associated with cell wall structure. The cell wall serves as a protective capsule that shelters nutrients from degradation and acts as a gatekeeper governing the bioaccessibility of nutrients to digestive enzymes [10], which highlights the regulatory role of cell walls in nutrient digestion and their metabolic fate. Based on the above evidence, we propose that particle size—as an indicator of cell wall structure and integrity—exerts a decisive influence on the nutritional characteristics of whole grain foods (WGFs), which is consistent with a research report on the influence of particle size on bread structure and postprandial glycemic, insulinemic and appetite responses in healthy adults [11]. Specifically, we hypothesize that intact cell walls within larger particles may selectively retain specific phytochemicals capable of stimulating GLP-1 synthesis. This study represents an attempt to establish a correlation between cell wall structure, phytochemical retention, and postprandial GLP-1 secretory responses.

Tartary buckwheat (*Fagopyrum tataricum* (L.) Gaertn.), a dicotyledonous pseudocereal, exhibits distinctive structural and nutritional traits that render it an ideal material for exploring cell wall functionality in grains. As a dicot species, its cell walls feature Type I architecture, consisting of roughly equivalent amounts of cellulose and cross-linking xyloglucans embedded within a pectic matrix [12]. This pectin-rich framework dictates key cell wall traits, such as porosity, charge density and microfibril spacing. Importantly, the non-blockwise de-esterification pattern of homogalacturonan within the pectic matrix enables the dispersion of endosperm cells under mechanical processing, which makes Tartary buckwheat an outstanding model to dissect structure–function associations in whole grains. Aside from its favorable structural characteristics, Tartary buckwheat is acknowledged as a medicinal plant with multiple bioactive benefits and a rich natural source of endogenous GLP-1 secretagogues [13]. In the present work, we investigated particle size-dependent digestive profiles of Tartary buckwheat including digestion kinetics and digestive metabolites, and their associations with GLP-1 secretion, with a central focus on how cell wall architecture modulates GLP-1 release. The outcomes of this study are anticipated to yield original mechanistic explanations for the health-promoting effects of whole grains and offer theoretical guidance for designing grain-derived functional foods.

## 2. Results

### 2.1. Characterization of Flour Particles

**Figure 1:**
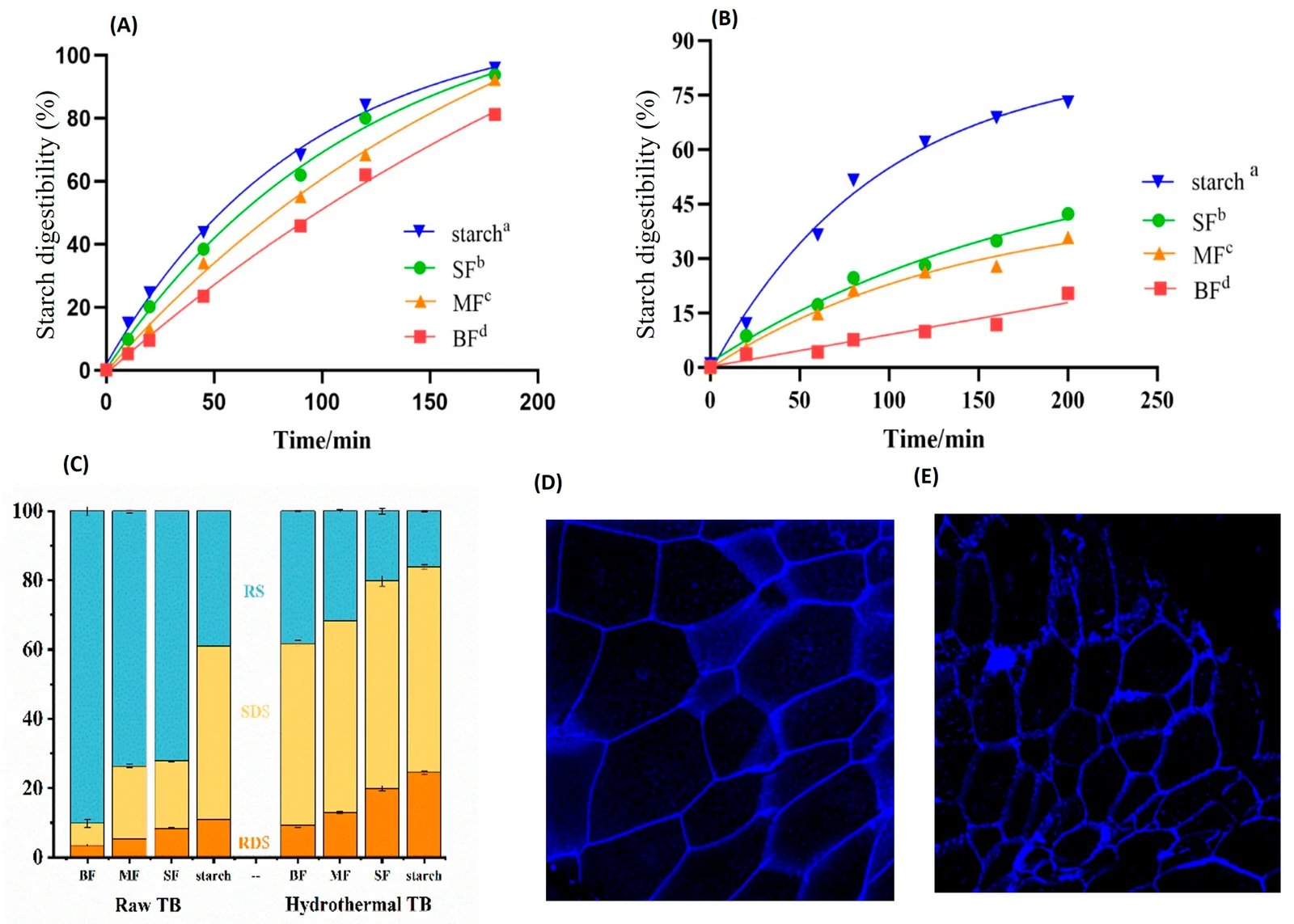
Particle size and starch digestion kinetics. Effect of dry-milled particle size on starch digestibility in raw TB (**A**), after hydrothermal treatment (**B**), the slow digestion property (**C**), and the fluorescence micrographs of cell walls in BF (**D**) and after thermal treatment (**E**). RS: resistant starch; SDS: slowly digestible starch; RDS: rapidly digestible starch. SF: small-sized particle fraction (0.3 mm in diameter); MF: middle-sized particle fraction (0.6 mm in diameter); BF: big-sized particle fraction (0.9 mm in diameter). Different letters represent significant difference at *p* < 0.05 between groups.

There are multiple components in grain kernels, and in order to investigate the influence of the cell-wall matrix structures on its nutritional property, confounding factors of the chemical compositions should be first eliminated. As there are different botanical layers in a grain kernel, pure endosperm with relatively uniform chemical composition and physical structure was used as the sample in this study. As shown in Table 1, the volume-median diameters (D50) were significantly different from each other, indicating the successful preparation of Tartary buckwheat (TB) flours with different particle sizes. More importantly, the prepared TB endosperm flours with different particle sizes exhibited similar proximate chemical composition as shown in Table 2, which satisfied the requirement of solely investigating the influence of physical structure under a similar chemical environment. Polyphenol (Table 2), the only component with significant difference among samples, might be a confounding factor, but the higher degree of starch digestion as shown for flour sample SF (0.2 mm in diameter, with the highest polyphenol value) when compared to other flour samples (Figure 1A,B) indicated that the inhibitory effect of polyphenols on starch digestion is likely masked by the large surface area of SF since particle surface area directly controls the bioaccessibility of digesting enzymes to their substrate, and the physical structure of cell wall (size and surface area) is the dominant factor modulating the digestion property of cell-wall-enclosed starch and other nutrients.

### 2.2. Cell Wall Intactness and Starch Digestion Properties

The TB was dry-milled to obtain flour particles with different sizes. When these particles were subjected to starch digestion process (Figure 1A), the mean percentage of digested starch were 84.09 ± 0.15% for starch, and 21.44 ± 0.06%, 35.79 ± 0.07%, 46.3 ± 0.08% for BF, MF, and SF, respectively, and all the endosperm particles exhibited a significant decrease in starch digestion when compared to purified starch, indicating that the cell wall matrix in the particles played a critical role in controlling starch digestion. However, when this difference was compared among uncooked particle samples, a similar degree of digestion, particularly at the end of the process, was observed, indicating that starch structure (semicrystalline structure) is another important factor influencing the rate and extent of starch digestion. Based on microscopic observation (Figure S1), larger particles (BF) are better able to maintain tissue structure during digestion, with only the outer cells being attacked during the digestion process and the inner cells remaining closely packed together. For flour sample MF, significant changes in cell morphology were observed in which some cell walls were difficult to observe, which is related to a higher digestion rate. Regarding the SF flour sample, starch granules, in the late stage of digestion, noticeably escaped from the cell walls and were rapidly digested. Consistently, this starch sample had the largest RDS content while the BF sample produced the largest RS (Figure 1C). Therefore, cell wall intactness in flour particles is the dominant factor controlling the rate and extent of starch digestion in endosperm particles, and the function of the cell wall matrix itself in maintaining tissue structure by providing cell–cell adhesion may also play a role in restricting cell expansion.

When the dry-milled and sieved particles were subjected to hydrothermal treatment (85 °C), broken edges in the cell wall (Figure 1D,E), indicating decreased intactness of the cell wall, and significant differences in the digestion kinetics of particles with different particle sizes were observed (Figure 1B). After 180 min, the difference in digestion degree between SF and MF particles and starch disappeared (91~96%), with only a lower level of digestion observed in BF particles (81.12 ± 0.37%). When the fractionation of starch was examined, particle size dependency was apparent for both RDS and RS. The RDS of all particles increased by almost three times after hydrothermal treatment while contrast changes were observed for RS that decreased from large to small particles. Although the contents of SDS among different particles were similar to each other (52~59%) (Figure 1C), an increase in RDS, which was converted from SDS after thermal treatment, also demonstrated the importance of particle size or cell wall intactness in controlling the slow digestion property of starch, which is similar with reports in the literature [14].

### 2.3. Postprandial GLP-1 Response to Isolated Buckwheat Endosperm Cells

**Figure 2:**
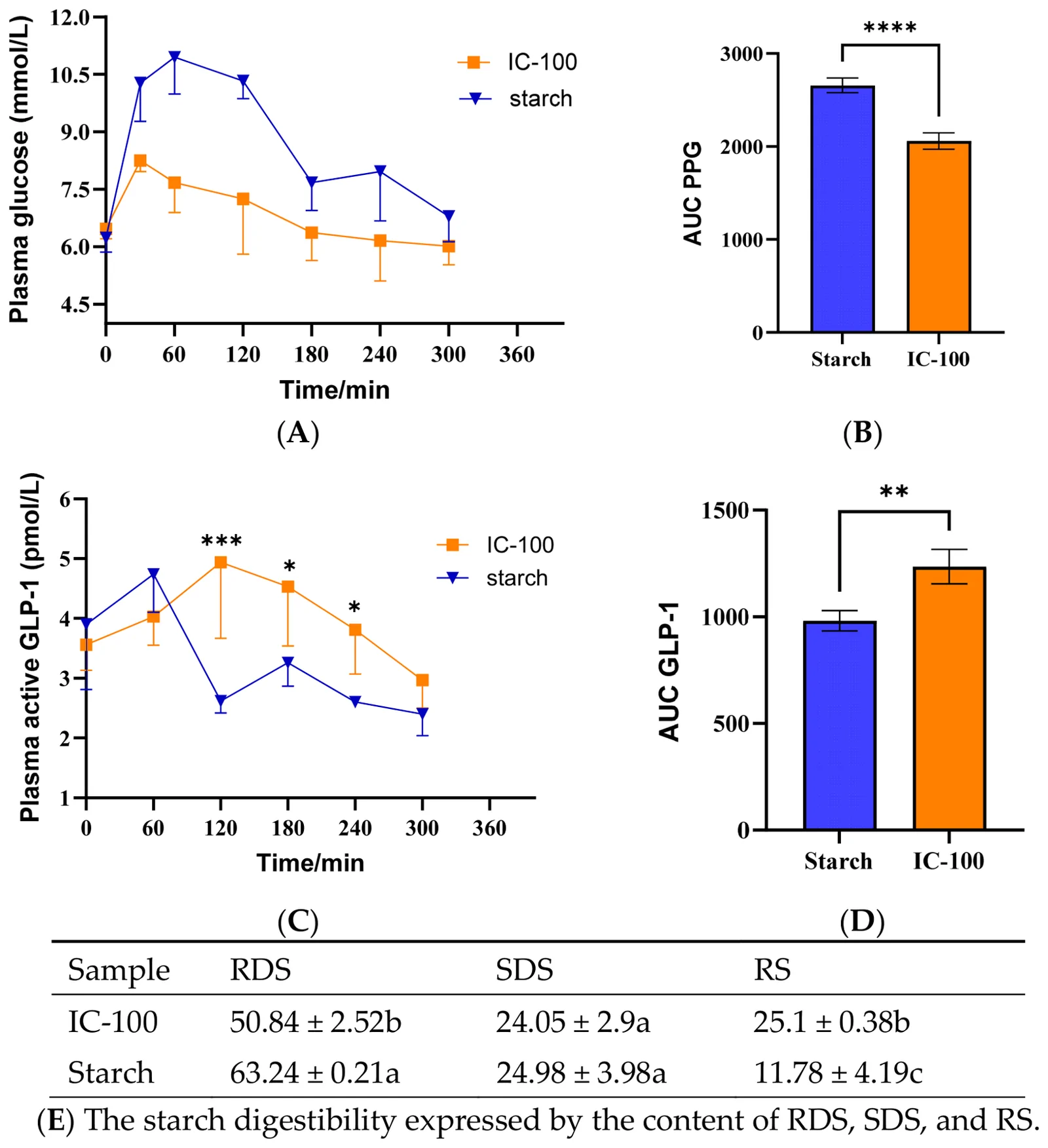
The postprandial glycemic response (**A**,**B**) and active GLP-1 response (**C**,**D**) after oral administration of cooked buckwheat starch and endosperm cells (IC-100) with different digestibility (**E**) in fasted mice. * represents a significant difference at *p* < 0.05, ** represents a significant difference at *p* < 0.01, *** represents a significant difference at *p* < 0.001, and **** represents a significant difference at *p* < 0.0001. Different letters represent significant difference at *p* < 0.05 (**E**).

GLP-1 is an incretin hormone secreted by intestinal L-cells in response to luminal nutrient stimulation, and an in vivo study using a mouse model was conducted to assess the relationship between the physical properties of the cell wall matrix and postprandial GLP-1 response. As shown in Figure 2, cooked starch (highest content in whole grain cells) induced a high level of postprandial glycemic response but markedly lower GLP-1 secretion while the cooked intact endosperm cells induced a significant and sustained GLP-1 release with flattened glycemic response, confirming that intact endosperm cells can not only stimulate GLP-1 secretion but also reduce the postprandial glycemic response, both of which are beneficial to blood glucose control. This result clearly demonstrates the superior health function of whole grain in regulating glucose homeostasis compared to cooked starch. Although various nutrient components in whole grain cells might produce more opportunities to stimulate L-cells, the cell wall in endosperm cells not only modulates the rate of digestion, but the digestion products might also be particle size-dependent, and the luminal nutrient stimulation from those digestion products is likely responsible for postprandial GLP-1 release.

### 2.4. GLP-1 Response to Buckwheat Flour Hydrolysate

**Figure 3:**
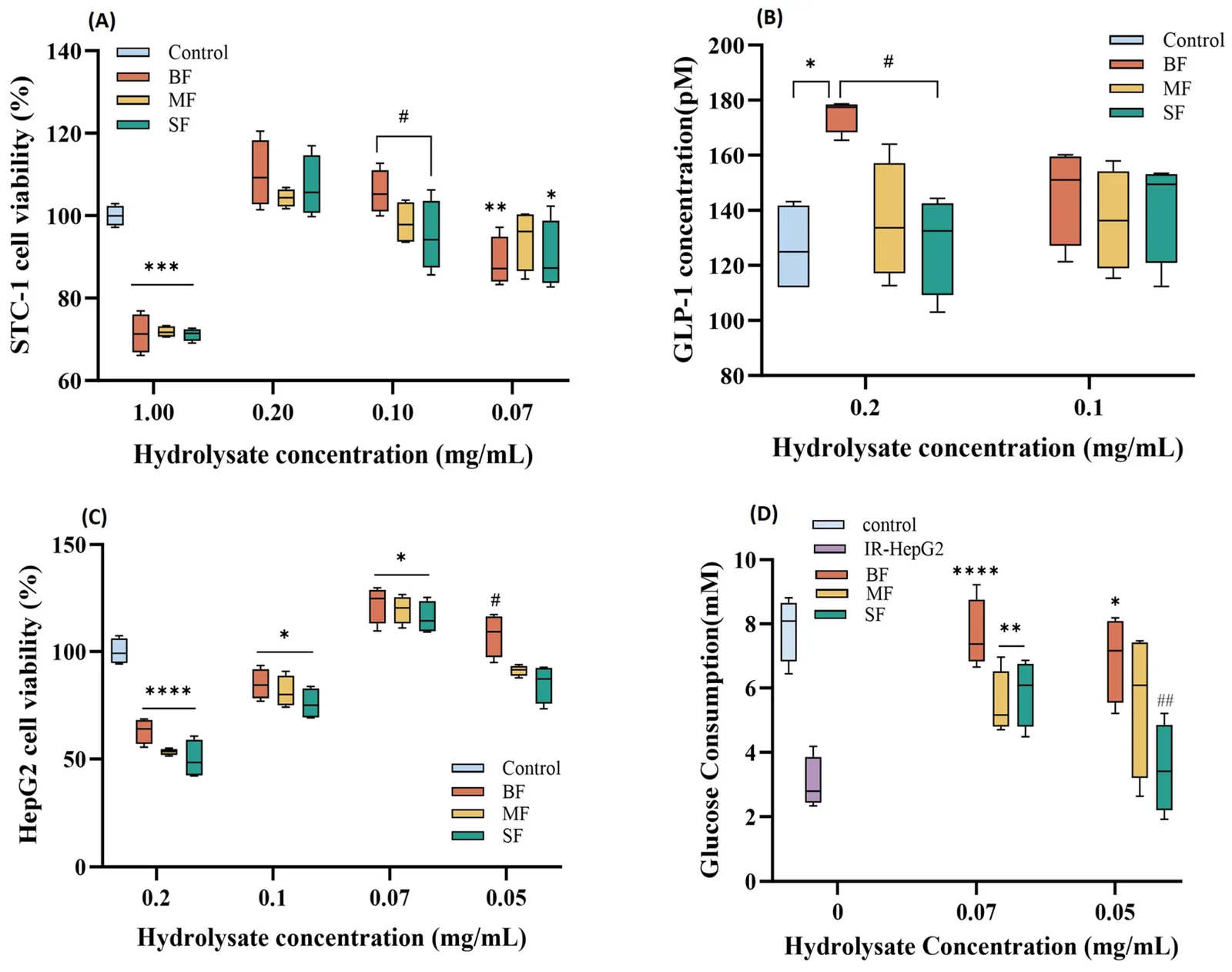
The viability of the L-cell line of STC-1 (**A**) and released GLP-1 content (**B**), and the glucose consumption of IRHepG2 cells (**C**,**D**) after treatment by hydrolysates from different samples. SF: small-sized particle fraction (0.3 mm in diameter); MF: middle-sized particle fraction (0.6 mm in diameter); BF: big-sized particle fraction (0.9 mm in diameter). * represents a significant difference at *p* < 0.05 when compared to the control, ** at a level of *p* < 0.01, *** at a level of *p* < 0.001, and **** at a level of *p* < 0.0001. # and ## represent significant differences within a group at *p* < 0.05 and *p* < 0.01, respectively. The relative concentration of hydrolysate is obtained by diluting the original hydrolysate (defined as 1 mg/mL) with culture medium.

Given the complex chemical composition of whole grains, the digestion-derived products that can stimulate GLP-1 secretion were investigated using the STC-1 L-cell model. Based on cell viability assays (Figure 3A), a hydrolysate concentration of 0.1–0.2 mg/mL was selected for GLP-1 secretion studies (Figure 3B). Notably, only the hydrolysate from the large particle-sized fraction (BF) significantly enhanced GLP-1 release (by ~40% vs. control) at 0.2 mg/mL, while hydrolysates from medium (MF) and small (SF) particle fractions had no observed effect at the same content of 0.2 mg/mL. This underscores particle size is a critical determinant of GLP-1-stimulatory activity. The cell viability data (Figure 3A) revealed an optimal hydrolysate concentration range that maintained STC-1 cell health, suggesting the presence of cytotoxic components at higher doses, which is consistent with prior reports on the toxic effect of phenolic compounds [15]. Intriguingly, GLP-1 secretion (Figure 3B) did not exhibit a classic dose–response relationship, implying that a synergistic balance of hydrolysate components may be necessary to trigger GLP-1 release. These findings highlight the need to identify the specific particle size-dependent components enriched in the BF hydrolysate, which aids the elucidation of the mechanistic link between grain microstructure and GLP-1 induction.

Glucose consumption, reflecting insulin sensitivity, was also assessed using an insulin-resistant HepG2 cells (IRHepG2). As shown in Figure 3C,D, the cell viability of HepG2 cells was also affected by the whole grain hydrolysate. At 0.07 mg/mL, the BF particle sample had the highest glucose consumption, indicating that the functional components in the hydrolysate of the BF sample can also increase the insulin sensitivity of IRHepG2 cells.

### 2.5. Bioinformatics Analysis of Digestion Products

**Figure 4:**
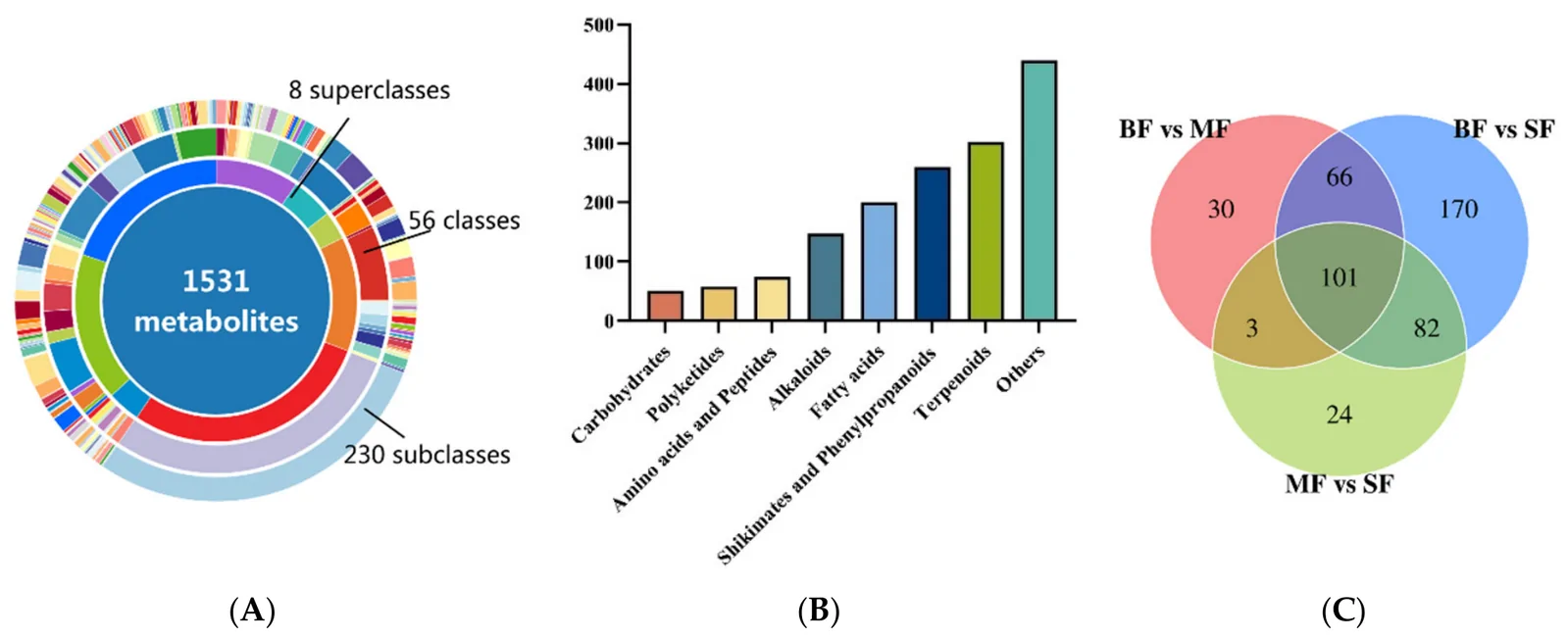
Overview of identified nutrient components from in vitro hydrolysate: donut plot of component classification (**A**), categories of nutrient components (**B**), and Venn diagram of differential digested products between groups (**C**).

To elucidate the mechanisms underlying the differential effects of hydrolysates on GLP-1 secretion, we employed a metabolomics approach to analyze the digested components following in vitro digestion. Our analysis identified 1531 distinct digestion products, which were categorized into eight major classes: carbohydrates, polyketides, amino acids, alkaloids, fatty acids, terpenoids, shikimates and phenylpropanoids, and others (Figure 4A). The Venn diagram revealed the greatest number of different digested products between the BF and SF groups, followed by BF and MF, and MF and SF. These findings not only highlight the remarkable compositional complexity of whole-grain digestion products but also provide a critical foundation for revealing the mechanistic link between particle size and GLP-1 secretory responses.

**Figure 5:**
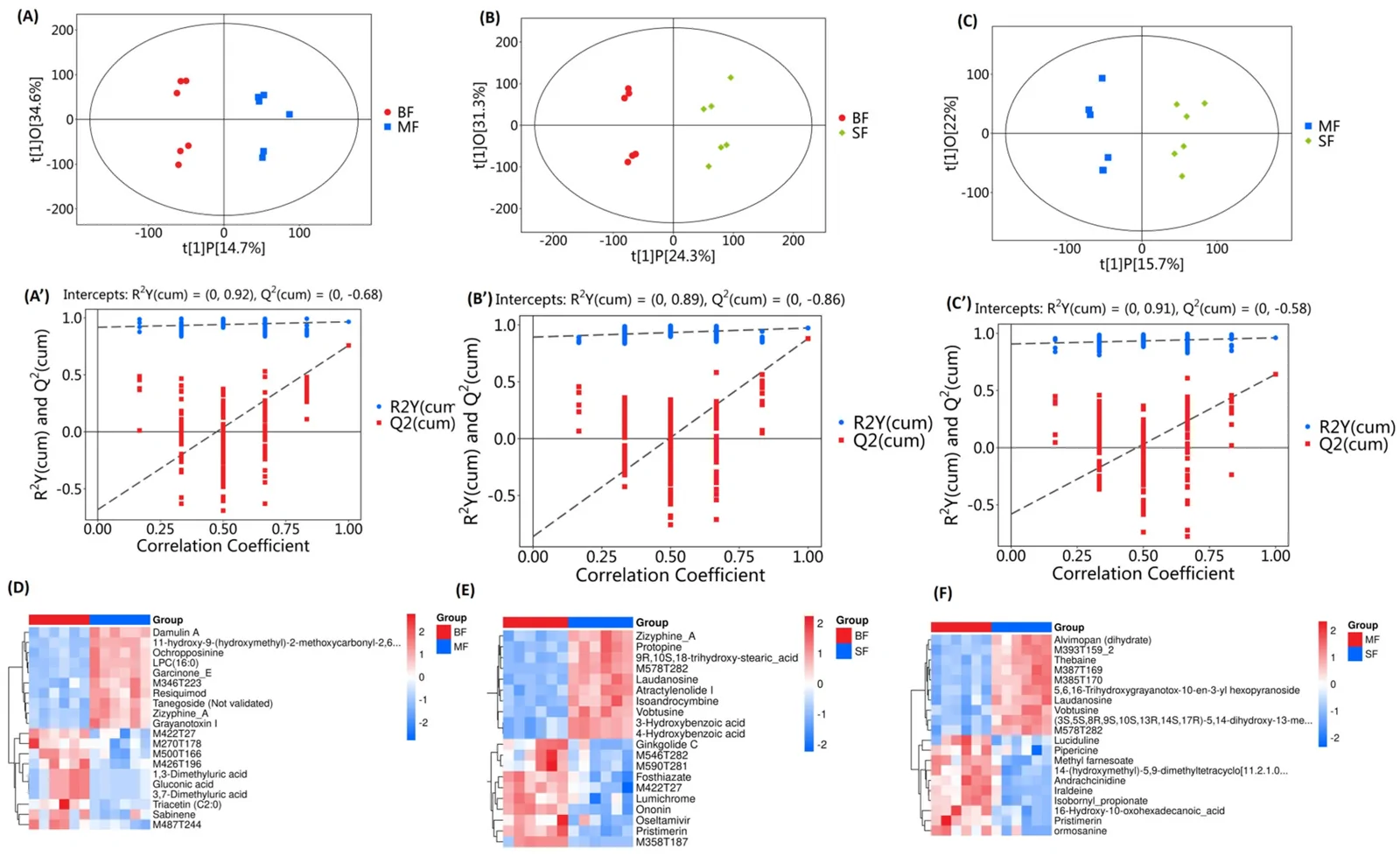
OPLS-DA analysis between groups with different particle sizes ((**A**) BF-MF, (**B**) BF-SF, and (**C**) MF-SF) and corresponding permutation plot test of OPLS-DA model (**A’**–**C’**). Comparison of digested product abundance between groups ((**D**) BF:MF, (**E**) BF: SF, and (**F**) MF-SF).

To test our hypothesis that particle size-dependent digestion alters the profiles of digested products in the hydrolysate, we analyzed differences in these profiles among groups using Orthogonal Partial Least Squares Discriminant Analysis (OPLS-DA). The OPLS-DA scatter plots (Figure 5A–C) revealed significant separation between groups, with all samples clustering within the 95% confidence interval. To ensure model robustness, we performed 7-fold cross-validation and permutation testing (Figure 5A’–C’). The high R^2^Y values (A: 0.965, B: 0.973, C: 0.961) confirmed the model’s accuracy in representing the sample data, while Q^2^ values > 0.40 (A: 0.758, B: 0.88, C: 0.642) indicated strong predictive capability for new samples. Together, these results demonstrated the model’s effectiveness in distinguishing sample groups. Further supporting this, a heatmap analysis of the top 10 significantly altered digested products (Figure 5D–F) showed clear differentiation among the BF, MF, and SF groups. Collectively, these findings confirm the particle size-dependent digestion properties of WGF samples in both digestion rate and hydrolysate composition, establishing a mechanistic basis for the observed differences in postprandial GLP-1 response and glucose consumption.

**Figure 6:**
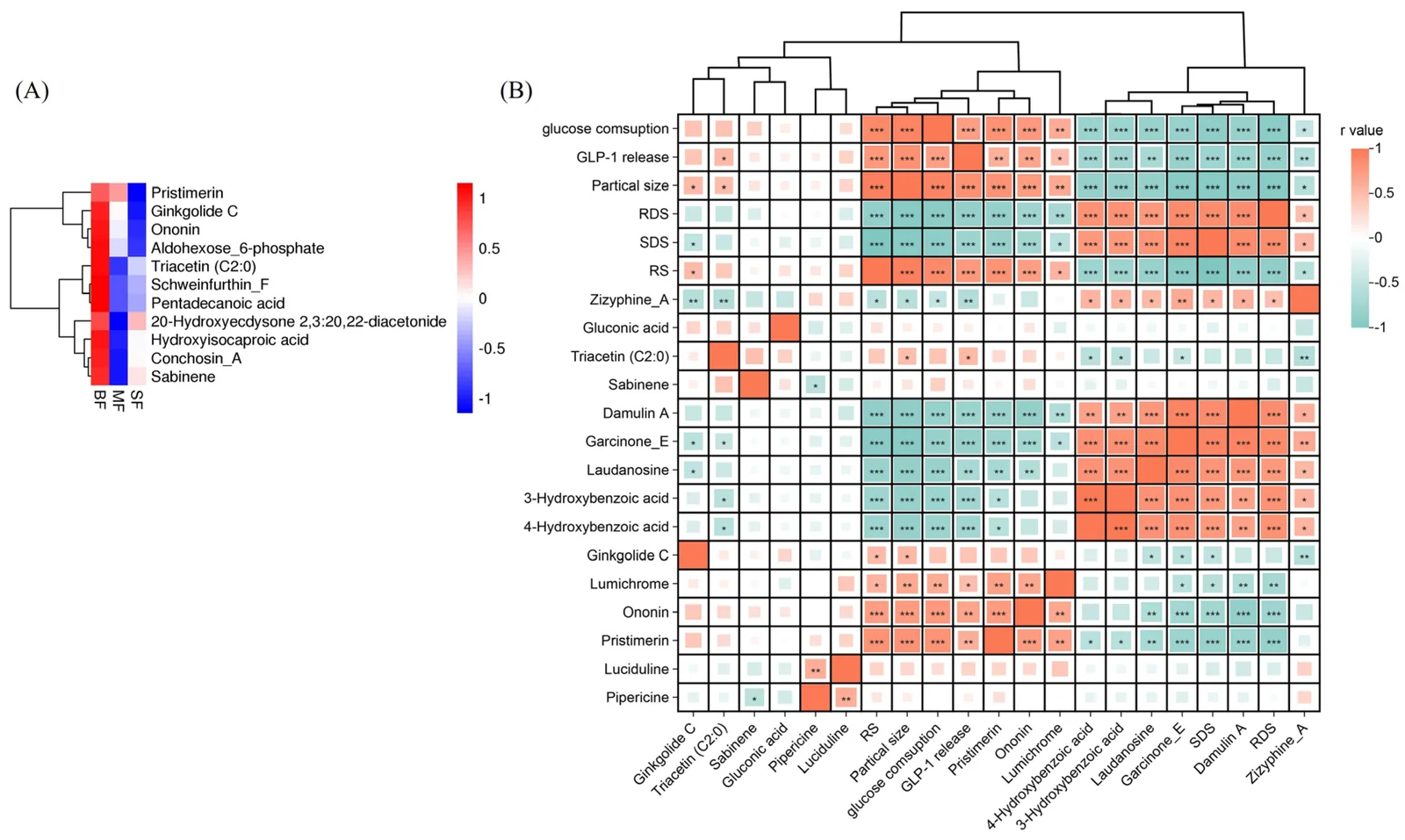
The heatmap of enriched components that are associated with GLP-1 release (**A**) in large particle sized fraction and the Spearman’s correlation analysis among the particle size, starch digestion parameters, GLP-1 release, glucose consumption and the bioactive compounds (**B**). Asterisks denote statistical significance: * *p* < 0.05, ** *p* < 0.01, and *** *p* < 0.001.

Among all digested products, we identified terpenoids (ginkgolide C, pristimerin, and conchosin-A), shikimates/phenylpropanoids (ononin), lipids (pentadecanoic acid, triacetin, and sabinene), carbohydrates (aldohexose-6-phosphate), stilbenoids (schweinfurthin-F), and phytoecdysteroids (20-hydroxyecdysone 2,3:20,22-diacetonide) as compounds significantly and positively correlated with GLP-1 release and glucose consumption. These phytochemicals were highly enriched in large particle-sized flour (BF) but present in lower amounts in MF and SF (Figure 6A). While the detailed mechanism of these phytochemicals in stimulating GLP-1 secretion and glucose consumption is not clear, their known bioactivities suggest plausible mechanisms: ginkgolide C can activate AMPK to reduce hepatic steatosis, fibrosis, and insulin resistance in NASH mice [16]; pentadecanoic acid (C15:0) promotes glucose uptake via the AMPK-AS160 pathway and exhibits insulin-sensitizing effects [16]; and pristimerin enhances intestinal barrier integrity and modulates gut microbiota [17], potentially linking it to GLP-1’s role in mitigating endothelial injury [18].

Correlation analysis (Figure 6B) revealed that ononin, ginkgolide C, pristimerin, triacetin, and pentadecanoic acid were positively correlated with GLP-1 release and resistant starch (RS) but negatively correlated with rapidly (RDS) and slowly digestible starch (SDS), indicating that RS, not SDS or RDS, is mechanistically linked to particle size and GLP-1 response. In the meantime, similar correlation between glucose consumption and other parameters was also observed, indicating these phytochemicals are the keys to both GLP-1 release and glucose consumption, which is the reason for the high correlation between GLP-1 and glucose consumption. Thus, phytochemicals enriched in large particles (high RS) likely drive GLP-1 secretion and insulin sensitivity improvement.

### 2.6. Molecular Docking on the Hydrolysate Metabolite-Mediated GLP-1 Secretion

**Figure 7:**
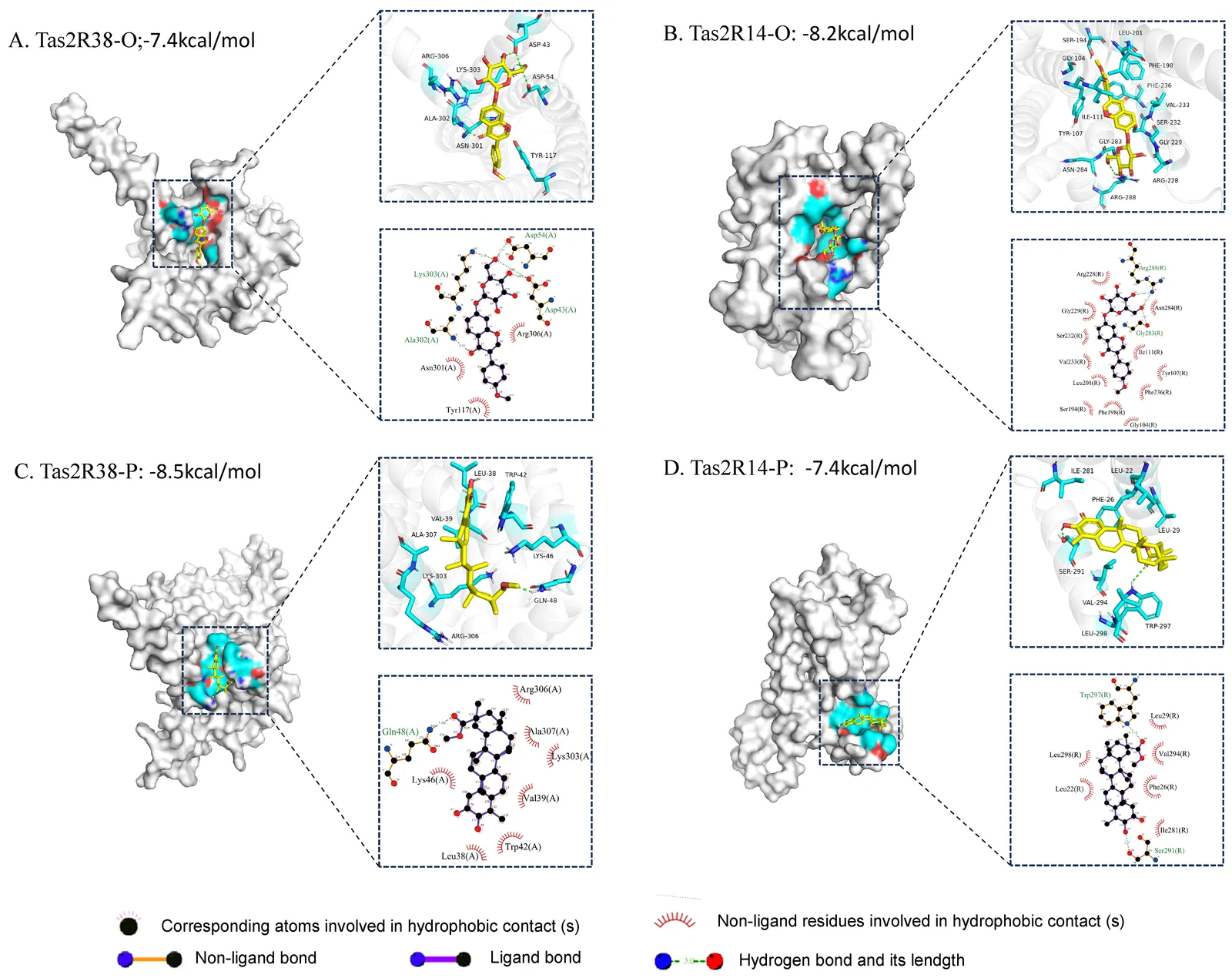

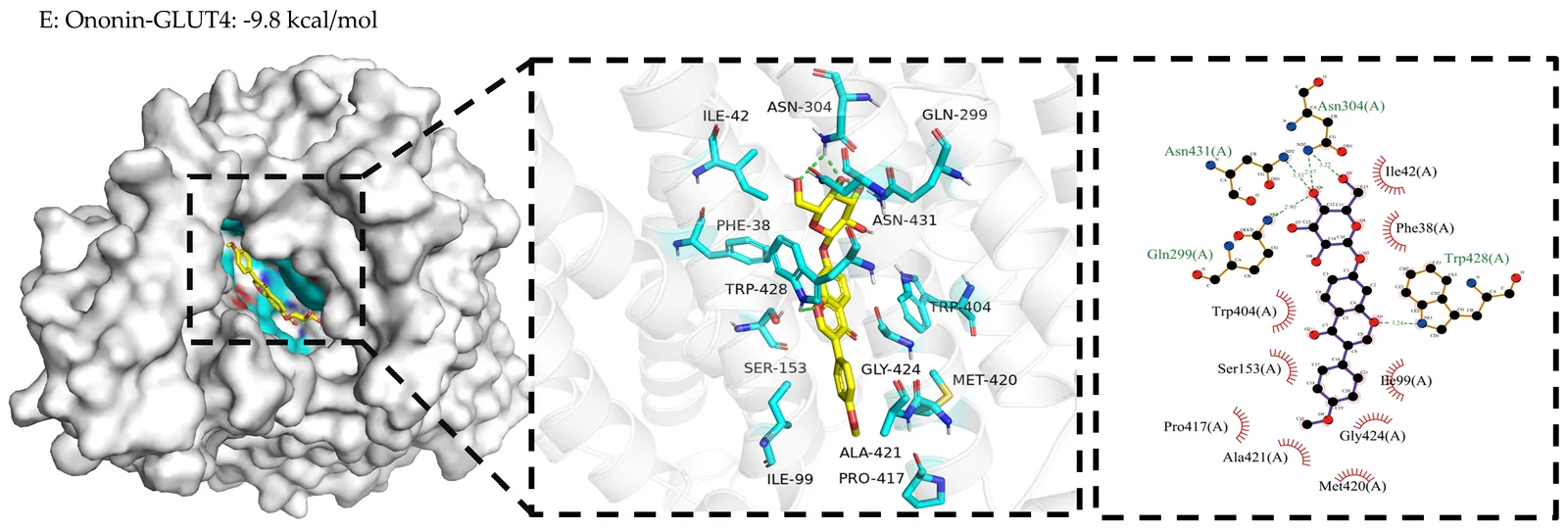
Molecular docking of ononin and pristimerin with bitter taste receptors of Tas2R38 and Tas2R14 as well as GLUT4. O: ononin; P: pristimerin.

To elucidate the mechanism by which cell wall-associated phytochemicals stimulate GLP-1 secretion, we employed molecular docking to analyze receptor–ligand interactions, binding modes, and affinities. Building on established findings that many bitter phytochemicals (e.g., tannins and berberine) activate intestinal bitter taste receptors (Tas2Rs) through the IP3/DAG pathway to induce GLP-1 secretion [19], we focused on two highly enriched metabolites from BF—ononin and pristimerin—docking them with Tas2R38 and Tas2R14 (Figure 7). The main binding sites of ononin are located at four hydroxyl groups on the pyranose ring (Lys303, Asp54, Asp43, and Ala302). The binding free energy of ononin was −7.4 kcal/mol and −8.2 kcal/mol for Tas2R38 and Tas2R14, respectively, indicating stronger binding affinity for these two receptors. 3D molecular simulation further reveals that ononin can be almost completely embedded into the hydrophobic pockets of Tas2R14. Although the binding affinity of ononin to Tas2R38 is relatively weaker than that to Tas2R14, more hydrogen bonds are formed between the ligand and protein residues. Specifically, the hydroxyl groups at the C7, C15 and C12 positions of ononin form five intermolecular interactions with LYS303, ASP43, ALA302 and ASP54, which stabilize the binding between the small molecule and Tas2R38. For pristimerin, the binding free energies with Tas2R38 and Tas2R14 are −8.5 kcal/mol and −7.4 kcal/mol, respectively, all forming stable ligand–receptor complexes. Nevertheless, the hydrophobic residues surrounding the pristimerin binding site on Tas2R14 differ markedly from those of ononin, suggesting distinct docking modes.

These findings indicate that different phytochemicals enriched in BF can activate distinct receptor targets, thereby stimulating GLP-1 secretion. Ren et al. [20] reported that bitter bioactive compounds in Banxia Xiexin Decoction, including baicalein, berberine, coptisine and kaempferol, possess strong binding affinity to Tas2R38 with key binding residues of LYS303 and ALA302, which is consistent with our results. BF-derived phytochemicals may differentially activate Tas2Rs to promote GLP-1 secretion through distinct molecular interactions. Regarding glucose consumption and insulin sensitivity, molecular docking between ononin and GLUT4 also showed strong binding (ΔG < −9.8 kcal/mol), and ononin might improve glucose absorption through modulating the activity of GLUT4 via the IRS1/PI3K/AKT/GLUT4 signaling pathway [21].

## 3. Discussion

Glucagon-like peptide-1 (GLP-1), a multifunctional gut hormone primarily secreted by intestinal L-cells in response to nutrient stimulation, has garnered significant interest due to its pleiotropic effects. Since its initial identification as a potent stimulator of insulin secretion and blood glucose regulation in clinical studies [22], GLP-1 receptor agonists (GLP-1RAs) have emerged as a cornerstone therapy for obesity and type 2 diabetes (T2DM) [23], with numerous drugs now available [24]. However, dietary strategies to enhance endogenous GLP-1 release represent a more sustainable approach for long-term health management. Whole grain flour, with its well-documented particle size-dependent slow digestion properties and ability to attenuate postprandial glycemic responses [25], may naturally become the resource to identify their potential function in GLP-1 secretion. Our study demonstrated that large particle-sized flour (BF) hydrolysates, enriched with specific bioactive phytochemicals, significantly enhanced GLP-1 release and glucose consumption from the cell studies. The in vivo postprandial responses to GLP-1 might also be related to these selectively enriched phytochemicals because of their luminal stimulation nature in the postprandial phase [26]. These findings suggest that GLP-1 induction may be a key mechanism underlying the health benefits of whole grains, consistent with reports showing that whole grain sorghum elevates postprandial GLP-1 levels and prolongs satiety in humans [1], and that millet polyphenols also boost endogenous GLP-1 production [2]. Importantly, since particle size reflects processing refinement, excessive grinding may compromise the health-promoting effects of whole grains. Thus, minimal processing [27,28] to preserve bioactive components appears optimal for maximizing their nutritional benefits.

The key novel finding of our study is the identification of GLP-1-stimulating phytochemicals that are selectively enriched in large-particle-size samples, confirming our hypothesis that digestion processes (slow or rapid) significantly influence both the profiles and metabolic fate of digested products. While most conventional nutrients (carbohydrates, amino acids, and fatty acids) showed increased concentrations with decreasing particle size, we observed distinct patterns for other compounds through K-means clustering analysis (Figure S2). These patterns included: (1) compounds decreasing along the BF → MF → SF gradient (including our identified bioactive phytochemicals); (2) compounds increasing from BF → MF but decreasing from MF → SF; and (3) compounds showing the opposite trend (decreasing then increasing). This complex behavior suggests that whole grain metabolism involves multiple kinetic pathways, where digestion timing critically determines metabolic fate. Specifically, rapidly released compounds from small particles may undergo enzymatic or non-enzymatic conversion, while large particles can protect bioactive phytochemicals with their cell walls in delayed digestion processes [29]. These findings highlight that optimizing whole grain processing to maintain larger particle sizes and intact cell wall structures could preserve bioactive phytochemicals, thereby enhancing GLP-1 response and other health benefits.

Our comprehensive analysis revealed that particle size showed significant positive correlations with many other parameters: GLP-1 production, resistant starch (RS) content, abundance of GLP-1-stimulating phytochemicals, and glucose consumption. The mechanism of phytochemical-induced GLP-1 secretion appears to involve multiple pathways, including binding to G protein-coupled receptors (particularly bitter taste receptors Tas2Rs), modulation of cAMP signaling in L-cells, and potential microbiota-mediated effects [8]. While slow digestion has traditionally been associated with postprandial GLP-1 response [30], our findings demonstrate that it is the RS content—not slowly digestible starch (SDS) or rapidly digestible starch (RDS)—that is positively correlated with GLP-1 release, suggesting that digested products rather than digestion kinetics drive this physiological response. This RS is likely formed through deep embedding within the cell wall matrix, revealing a novel mechanism by which dietary fiber (via cell wall architecture) modulates the health benefits of whole grain foods through the selective retention and protection of specific bioactive compounds. These findings also highlight the complex interaction (binding and retention) between phytochemicals and cell-wall polysaccharides [6] in food processing, which is crucial for the enhanced health benefits of WGFs [6].

## 4. Materials and Methods

### 4.1. Characterization of Flour Particles

Dehulled Tartary buckwheat (*Fagopyrum tataricum* (L.) Gaertn.) seeds (8.5% moisture) were conditioned to 12% moisture (25 °C, 24 h) and processed using a degerminator (MHXM, Bühlergroup, Uzwil, Switzerland; 1500 rpm, 50 g/min feed rate) to obtain >95% pure endosperm (verified by Calcofluor White staining). The endosperm was milled (Perten 3100, Hägersten, Sweden, 20 °C, three passes) and sieved (Retsch AS200, Haan, Germany, 15 min) into three fractions, namely, big size fraction (BF: 0.9 mm/20 mesh, 42% yield), middle size fraction (MF: 0.6 mm/30 mesh, 38%), and small size fraction (SF: 0.2 mm/70 mesh, 20%), with particle sizes confirmed by laser diffraction. Native starch was isolated via alkaline extraction (AACC 76-13.01; 98.2% purity, <0.3% protein). Reagents included Calcofluor White M2R (Sigma F3543, St. Louis, MO, USA), a glucose HK-kit (Sigma GAHK-20, St. Louis, MO, USA), a total starch assay (Megazyme K-TSTA, Bray, Ireland), and a GLP-1 ELISA kit (Nanjing Jiancheng H203, Nanjing, China, sensitivity 0.5 pmol/L).

### 4.2. Particle Characterization and Chemical Composition Analysis

Flour particle size distribution was analyzed in triplicate using a laser diffraction particle size analyzer (Microtrac S3500, Montgomeryville, PA, USA) with dry dispersion (0.5 bar pressure, 50% feed rate), reporting volume median diameter (D50) and polydispersity (span = [D90 − D10]/D50) according to ISO 13320:2020 as described in the literature [31]. The total starch content was quantified using the Megazyme Total Starch Assay Kit (K-TSTA) following the manufacturer’s protocol (AOAC Method 996.11). Proximate composition analysis was performed according to AACC International Approved Methods (2009): moisture content (Method 44-15.02), crude protein (Method 46-30.01; N × 5.83), and total fat (Method 30-25.01). Total phenolic content was determined spectrophotometrically using the Folin–Ciocalteu method with gallic acid as standard [32], while total flavonoids were measured by the aluminum chloride colorimetric method [33] with catechin equivalents. All analyses were conducted in triplicate on dried samples (lyophilized for 48 h), with results expressed on a dry weight basis (mean ± SD, n = 3).

### 4.3. Starch Digestion Kinetics

Enzymatic hydrolysis was employed to characterize the digestion kinetics of raw and hydrothermally treated (85 °C, 20 min with intermittent stirring) Tartary buckwheat flours (SF: 0.2 mm; MF: 0.6 mm; BF: 0.9 mm) and isolated starch controls. Samples (100 ± 5 mg starch equivalents) were suspended in 10 mL sodium acetate buffer (0.1 mol/L, pH 5.2) and digested using α-amylase (290 U/mL) and glucoamylase (15 U/mL) at 37 °C with continuous shaking (160 rpm). Aliquots were collected at timed intervals, with reactions terminated by 80% ethanol addition. Glucose release was quantified using a hexokinase assay kit (HK-kit), and hydrolysis curves were fitted to first-order kinetics:

(1) C_t_ = C_∞_(1 − e^−kt^)

where C_t_ is digestible starch (%) at time t, C_∞_ is equilibrium concentration, and k is the digestion rate coefficient.

To differentiate factions of starch with different digestibilities, total starch content (TS) and the concentration of free glucose in the starch (FG) after 20 min (G20) and 120 min (G120) of digestion were calculated and recorded: rapidly digestible starch (RDS = [(G20 − FG) × 0.9]/TS × 100), slowly digestible starch (SDS = [(G120 − G20) × 0.9]/TS × 100), and resistant starch (RS = [TS − (RDS + SDS)]/TS × 100).

### 4.4. Confocal Microscopic Observation

Samples were fixed overnight in modified Karnovsky’s fixative (1.6% *v*/*v* formaldehyde and 2% *v*/*v* glutaraldehyde in 0.1 M phosphate buffer, pH 7.4) at 4 °C, then cryosectioned into 5–10 μm thick sections using a freezing ultramicrotome (Leica CM1950, Wetzlar, Germany) with chamber temperature maintained at −20 °C. Sections were stained with 0.1% Calcofluor White M2R (Sigma F3543, St. Louis, MO, USA) in PBS for 2 min, rinsed three times with distilled water, and mounted with antifade medium. Imaging was performed using a confocal laser scanning microscope (Zeiss LSM 880, Oberkochen, Germany) equipped with a 405 nm diode laser for excitation and a 420–480 nm bandpass emission filter, with all images captured using a 40× oil immersion objective (NA 1.3) and standardized acquisition parameters (laser power 5%, gain 800, pixel dwell 1.58 μs).

### 4.5. Animal Experiment Protocol

All animal procedures were approved by the Jiangnan University Animal Ethics Committee (Approval No. JN.20240229b0760530[051]) and conducted in compliance with the National Research Council’s Guide for the Care and Use of Laboratory Animals (8th edition). Twenty mice (18–22 g) were acclimatized for 7 days in a controlled environment (22–26 °C, 40–60% humidity, 12 h light/dark cycle with lights on at 06:00) with *ad libitum* access to standard chow and water. Following overnight fasting, mice were randomly divided into two groups (n = 10/group) and administered either: (1) cooked buckwheat starch or (2) intact endosperm cells (2 g/kgbw by oral gavage, IC-100: intact cells, 100–125 µm). The gavage sample (1.0 g starch or endosperm cell in 10 mL deionized water) was pretreated in a water bath at 85 °C for 20 min. To prevent GLP-1 degradation, sitagliptin (25 mg/kg body weight (kgbw) in saline) was first administered orally based on its pharmacokinetic profile (t1/2 < 2 h). Blood samples (40 μL) were then collected via saphenous vein puncture into EDTA-coated tubes containing DPP-4 inhibitor cocktail at 0 (baseline), 30, 60, 90, 120, 180, 240, and 300 min post-dosing. Plasma was immediately separated by centrifugation (4000× *g*, 10 min, 4 °C) and stored at −80 °C until GLP-1 quantification using a commercial ELISA kit (intra-assay CV < 8%, inter-assay CV < 12%).

Tartary buckwheat seeds were gently ground in a mortar and sieved through a 200-μm mesh to remove hulls, seed coats, and cotyledons. The resulting flour was subsequently sieved through 125 μm and 100 μm meshes in sequence. The flour fraction that passed through the 125 μm mesh but was retained on the 100 μm mesh was washed with a fine water spray. The retained material was examined under a microscope to determine its cellular structure and was regarded as intact endosperm cells.

### 4.6. In Vitro Gastrointestinal Digestion

The INFOGEST 2.0 protocol [34] was adapted to simulate gastric and intestinal phases without oral processing. Sample porridges were prepared by homogenizing 1.0 g of each buckwheat flour fraction (SF, MF, and BF) with 10 mL purified water, boiling at 100 °C for 20 min (with stirring every 5 min), and equilibrating to 37 °C for 20 min. Gastric digestion was initiated by adding simulated gastric fluid (SGF) containing pepsin (2000 U/mL final activity, pH 3.0) and incubating at 37 °C with 60 rpm horizontal shaking for 60 min. The intestinal phase was initiated by adding simulated intestinal fluid (SIF) containing pancreatin (100 U/mL trypsin activity) and bile salts (10 mM), with continued incubation under identical conditions for 180 min. Aliquots (500 μL) were collected at 0, 30, 60, 80, 100, 130, 160, and 180 min, immediately mixed with 1 mL ice-cold absolute ethanol to terminate enzymatic activity, and centrifuged (10,000× *g*, 10 min, 4 °C). Released glucose in supernatants was quantified using a hexokinase/glucose-6-phosphate dehydrogenase assay (HK-kit, Sigma), with starch digestion rates calculated from standard curves and expressed as percentage hydrolysis relative to total starch content. All digestions were performed in triplicate with appropriate enzyme-free controls.

### 4.7. Metabolomics Analysis of the Hydrolysates from Different Particle-Sized Samples

Following INFOGEST 2.0 in vitro digestion, enzymatic reactions were terminated by heat inactivation (100 °C, 5 min) and samples were centrifuged (12,000× *g*, 10 min, 4 °C). For metabolite extraction, 100 μL aliquots of supernatant were combined with 400 μL of ice-cold extraction solvent (methanol: acetonitrile, 1:1 *v*/*v*) containing stable isotope-labeled internal standards (10 ppm each of d4-TSP, 13C6-sorbitol, and d9-cholic acid). The mixture was vortexed vigorously (30 s), sonicated at −40 °C (1 h, 40 kHz), and centrifuged (15,000× *g*, 15 min, 4 °C). The supernatant was filtered through a 0.22 μm PTFE membrane prior to UHPLC-Orbitrap MS analysis. Chromatographic separation was achieved using a Phenomenex Kinetex C18 column (2.1 × 50 mm, 2.6 μm) with mobile phase A (0.01% acetic acid in water) and B (isopropanol: acetonitrile, 1:1 *v*/*v*) at 0.3 mL/min flow rate with the following gradient: 0–2 min (5% B), 2–15 min (5–95% B), 15–18 min (95% B), 18–18.1 min (95–5% B), 18.1–20 min (5% B). The Orbitrap Exploris 120 mass spectrometer operated in both positive and negative ionization modes with the following parameters: spray voltage ± 3.5 kV, capillary temperature 320 °C, sheath gas 40 arb, aux gas 10 arb, full scan range *m*/*z* 50–1500 at 120,000 resolution, and data-dependent MS/MS (top 5 ions, HCD collision energy 30 eV). Data acquisition and processing were performed using Xcalibur software (v4.4, Thermo Scientific) with quality controls injected every 10 samples.

### 4.8. STC-1 Cell Viability and Secretion of GLP-1

STC-1 enteroendocrine cells (BNCC342403, BeNa Culture Collection, Beijing, China) were maintained in high-glucose DMEM (BNCC338068, BeNa Culture Collection, Beijing, China) supplemented with 10% FBS and 1% penicillin–streptomycin at 37 °C under 5% CO_2_. For viability assays, cells were seeded in 96-well plates (5 × 10^4^ cells/well, n = 6 replicates/group) and allowed to adhere overnight. Following 12 h exposure to hydrolysates (0.1–0.2 mg/mL), cell viability was assessed using CCK-8 reagent (Beyotime, Shanghai, China) per manufacturer’s protocol: absorbance was measured at 450 nm (reference 650 nm) after 120 min incubation, with viability normalized to untreated controls. For GLP-1 secretion studies, cells were plated in 24-well plates (3 × 10^5^ cells/well, n = 3) and serum-starved for 1 h in HEPES buffer (pH 7.4, containing 0.1% BSA) prior to 12 h treatment with hydrolysates. Supernatants were collected after centrifugation (300× *g*, 5 min) and stored at −80 °C until analysis. Active GLP-1 levels were quantified using a commercial ELISA kit (Nanjing Jiancheng; Nanjing, China, sensitivity 0.5 pM, intra-assay CV < 8%), with all samples analyzed in duplicate and values corrected for total cellular protein (BCA assay, Beyotime, Shanghai, China).

### 4.9. Glucose Consumption and Insulin Sensitivity of HepG2 Cells

HepG2 cells were cultured in DMEM medium (BNCC338068) in a cell incubator at 37 °C with 5% CO_2_, and subculture was conducted when the cell confluency reached 80%. The insulin-resistant HepG2 (IR-HepG2) cell model was established by culturing the cells for 72 h in a medium containing 30 mmol/L glucose and 1 µmol/L insulin. After washing with PBS, the cells were further cultured in normal medium for an additional 6 h. The glucose content in the supernatant of the cell culture medium after centrifugation was determined to verify the successful establishment of the IR-HepG2 model. The glucose consumption of the cells was calculated by subtracting the glucose content in the supernatant. Subsequently, the established IR-HepG2 model cells were incubated for 6 h in a medium containing 1 µM insulin and 30 mmol/L glucose as well as the digestive products from Tartary buckwheat flour samples, and the supernatant was collected to measure the glucose consumption to reflect the insulin sensitivity of the IR-HepG2 influenced by Tartary buckwheat flour hydrolysates.

### 4.10. Molecular Docking

Protein–ligand interactions were predicted using AutoDock Vina (version 1.2.7) with the following workflow [20]: (1) Receptor preparation: Tertiary structures of bitter taste receptors Tas2R38 (AlphaFold DB: AF-P59533-F1), Tas2R14 (PDB ID: 9IJA) were processed in PyMOL 2.5 (Schrödinger) by removing crystallographic water molecules and non-native ligands, followed by protonation state adjustment at pH 7.4 using AutoDockTools 1.5.6. (2) Ligand preparation: Phytochemical 3D structures (ononin: CID 442813, ginkgolide C: CID 161582, pristimerin: CID 159516) were retrieved from PubChem, energy-minimized (MMFF94 force field), and converted to PDBQT format using OpenBabel 3.1.1. (3) Docking parameters: Grid boxes (30 × 30 × 30 Å) centered on known binding pockets were generated with 0.375 Å spacing. (4) Simulation: In total, 20 independent runs per ligand–receptor pair were performed using Lamarckian genetic algorithm (population size: 150, maximum evaluations: 2.5 × 10^6^). (5) Analysis: The lowest energy conformations were validated by root-mean-square deviation (RMSD) clustering (<2.0 Å), with intermolecular interactions analyzed in UCSF Chimera 1.16 and visualized using PyMOL 2.5/LigPlot+ v.2.2.

### 4.11. Statistical Analysis

Data were analyzed using a one-way ANOVA with Tukey’s honestly significant difference (HSD) post hoc test for multiple comparisons (α = 0.05), implemented in GraphPad Prism 10.0 (GraphPad Software, San Diego, CA, USA) and SPSS 26.0 (IBM Corp., Armonk, NY, USA). Normality was verified using Shapiro–Wilk tests (*p* > 0.05) and homogeneity of variance with Levene’s test. Multivariate analyses included: (1) Orthogonal Partial Least Squares-Discriminant Analysis (OPLS-DA) with 7-fold cross-validation, (2) Venn diagrams to identify unique/overlapping metabolites, (3) heatmap visualization of z-score normalized data (Euclidean distance, complete linkage), and (4) Spearman’s rank correlation (ρ) for non-parametric relationships. All bioinformatics analyses were performed in R 4.2.2 (R Foundation, Vienna, Austria) using the ropls, VennDiagram, pheatmap, and corrplot packages, respectively. Effect sizes are reported as partial eta-squared (ηp^2^) for ANOVA and 95% confidence intervals for post hoc tests.

## 5. Conclusions

This study investigated how the cell wall matrix structure of whole grain buckwheat influences postprandial GLP-1 responses and glucose absorption, revealing that intact endosperm cells (abundant in large particle sizes) enhance GLP-1 secretion primarily through selectively enriched bioactive phytochemicals in the cell wall matrix. This highlights a novel function of dietary fiber (in its physical cell wall form): beyond modulating digestion kinetics, it may selectively retain and protect phytochemicals, thereby amplifying their bioactivity. These results significantly advance our understanding of cell wall functionality, providing direct evidence of synergistic interactions between dietary fiber and phytochemicals—the two major bioactive components in whole grain foods (WGFs)—that collectively mediate their health benefits. Importantly, since the physical structure of the cell wall is fundamental to this process, optimizing food processing (such as minimal milling) to preserve intact cell wall architecture is likely a critical strategy in maximizing the health-promoting effects of WGFs.

## Figures and Tables

**Table 1:** The particle size of buckwheat flours.

Flour	Particle Size			
	D_50_/μm	D (3, 2)	D (4, 3)	Span
BF	1143.3 ± 28.19 ^a^	1100.3 ± 71.23 ^a^	1124.4 ± 93.61 ^a^	0.5 ± 0.02 ^b^
MF	884 ± 10.03 ^b^	859.6 ± 36.67 ^b^	872.4 ± 24.02 ^b^	0.4 ± 0.05 ^b^
SF	432.4 ± 4.69 ^c^	395.8 ± 18.43 ^c^	510.2 ± 45.35 ^c^	1.4 ± 0.27 ^a^

Note: Different letters represent significant difference (*p* < 0.05) between samples.

**Table 2:** The chemical compositions of TB flours with different particle sizes (d.w.-based percentages).

Flour	Chemical Composition							
	Water Content (%)	Total Starch (%)	Protein (%)	Fat (%)	Hemi- Cellulose (%)	Cellulose (%)	Flavone (mg/g)	Polyphenol (mg/g)
BF	11.26 ± 0.06	71.08 ± 2.66	2.33 ± 0.03	3 ± 0.08	6.5 ± 0.15	7.52 ± 0.5	4.88 ± 0.07	6.29 ± 0.05 ^b^
MF	11.34 ± 0.52	72.82 ± 1.21	2.12 ± 0.07	3.44 ± 0.02	6.9 ± 0.12	6.96 ± 1.16	4.34 ± 0.31	6.24 ± 0.12 ^b^
SF	10.59 ± 0.58	74.23 ± 1.34	2.34 ± 0.02	3.27 ± 0.07	6.25 ± 0.3	7.41 ± 0.45	4.28 ± 0.04	7.32 ± 0.32 ^a^

Note: Different letters represent significant difference (*p* < 0.05) between samples.

## Data Availability

The original contributions presented in this study are included in the article. Further inquiries can be directed to the corresponding author.

## Supplementary Materials

The following supporting information can be downloaded at: https://www.mdpi.com/article/10.3390/ijms27219470/s1.
