# Supplementary material for: Cell Wall-Formed Dietary Fiber in Tartary Buckwheat Governs Selective Enrichment of Phytochemicals to Enhanced Postprandial GLP-1 Response
Source: Int J Mol Sci. 2026 Oct 23;27(21):9470. doi: 10.3390/ijms27219470 (PMC13763364; doi:10.3390/ijms27219470)
Supplement: Supplementary file 1 [file ijms-27-09470-s001.zip › ijms-4587163-supplementary.pdf]

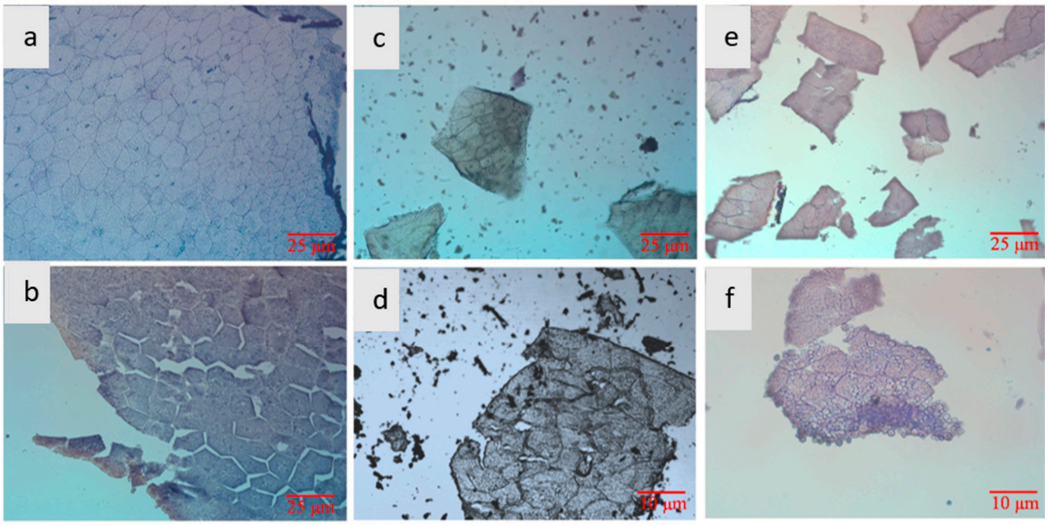

Figure S1. Digestion of uncooked flour particles. Light micrographs of BF (a and b), MF (c and d), and SF (e and f) captured before (a, c, e) and after (b, d, f) digestions.

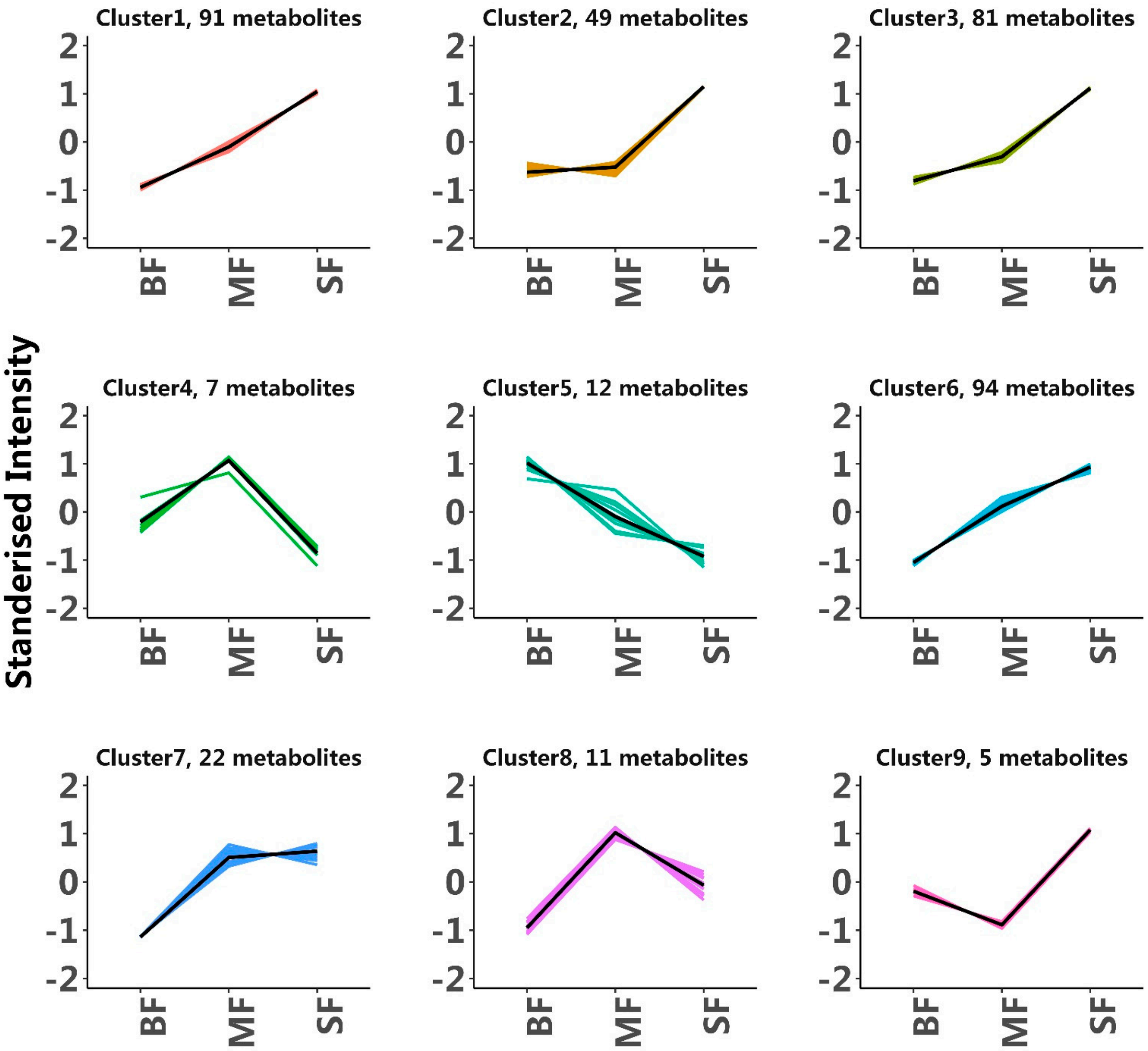

Figure S2. The K-Means analysis. BF, MF and SF are flour fractions with big, middle and small particle size.
